# Happiness Meanders: A cross-cultural dataset on happiness, emotions, and models of selfhood from 48 countries

**DOI:** 10.1038/s41597-026-07175-6

**Published:** 2026-05-13

**Authors:** Kuba Krys, Julien Teyssier, Valentin El Sayed, Olga Magdalena Gajewska, Agata Kocimska-Bortnowska, Aleksandra Kosiarczyk, Iva Poláčková Šolcová, Espen Røysamb, Eric Raymond Igou, Stanislava Stoyanova, Fridanna Maricchiolo, John M. Zelenski, Christin-Melanie Vauclair, Yukiko Uchida, David Sirlopú, Charity Akotia, Isabelle Albert, Lily Appoh, Douglas Marlon Arévalo Mira, Arno Baltin, Patrick Denoux, Alejandra Domínguez Espinosa, Carla Sofia Esteves, Vladimer Gamsakhurdia, Alin Gavreliuc, Diana Boer, Natalia Kascakova, Lucie Klůzová Kračmárová, Olga Kostoula, Nicole Kronberger, Anna Kwiatkowska, Hannah Lee, Magdalena Łużniak-Piecha, Arina Malyonova, Pablo Eduardo Barrientos Marroquin, Tamara Mohorić, Oriana Mosca, Elke Murdock, Nur Fariza Mustaffa, Martin Nader, Azar Nadi, Ayu Okvitawanli, Yvette van Osch, Vassilis Pavlopoulos, Zoran Pavlović, Muhammad Rizwan, Vladyslav Romashov, Rūta Sargautytė, Beate Schwarz, Heyla Selim, Ursula Serdarevich, Maria Stogianni, Chien-Ru Sun, Wijnand van Tilburg, Claudio Torres, Vivian L Vignoles, Cai Xing, Liman Man Wai Li, Vivian Miu-Chi Lun, Michael Harris Bond, June Chun Yeung, Brian W. Haas, Joonha Park, Mladen Adamovic, Ragna Benedikta Gardarsdottir, David Igbokwe, İdil Işık, Aleksandra Buźniak, Márta Fülöp

**Affiliations:** 1 Institute of Psychology, Polish Academy of Sciences, Warsaw, Poland; 2 Université Toulouse Jean Jaurès, Toulouse, France; 3 SWPS University, Wrocław, Poland; 4 Czech Academy of Sciences, Prague, Czechia; 5 University of Oslo, Oslo, Norway; 6 University of Limerick, Limerick, Republic of Ireland; 7 Department of Psychology, South-West University “Neofit Rilski”, Blagoevgrad, Bulgaria; 8 University of Roma Tre, Rome, Italy; 9 Carleton University, Ottawa, Canada; 10 Instituto Universitário de Lisboa, Lisboa, Portugal; 11 Kyoto University, Kyoto, Japan; 12 Faculty of Communication, History, and Social Sciences, Universidad Católica de la Santísima Concepción, Concepción, Chile; 13 School of Social Sciences, University of Ghana, Accra, Ghana; 14 University of Luxembourg, Esch-sur-Alzette, Luxembourg; 15 Nord University, Bodø, Norway; 16 HULAB, Comprometidos con tu desarrollo, San Salvador, El Salvador; 17 Tallinn University, Tallinn, Estonia; 18 Iberoamerican University, Mexico City, Mexico; 19 Católica Lisbon School of Business and Economics, Lisbon, Portugal; 20 Ivane Javakhishvili Tbilisi State University, Tbilisi, Georgia; 21 West University of Timisoara, Timisoara, Romania; 22 University of Koblenz, Koblenz, Germany; 23 Palacky University, Olomouc, Czechia; 24 Johannes Kepler University Linz, Linz, Austria; 25 Institute of Psychology, Johannes Kepler University Linz, Linz, Austria; 26 Indiana University Northwest, Gary, USA; 27 Dostoevsky Omsk State University, Omsk, Russia; 28 Universidad del Valle de Guatemala, Ciudad de Guatemala, Guatemala, Guatemala; 29 University of Rijeka, Rijeka, Croatia; 30 University of Cagliari, Cagliari, Italy; 31 International Islamic University Malaysia, Kuala Lumpur, Malaysia; 32 Universidad ICESI, Cali, Colombia; 33 Universitas Sebelas Maret, Surakarta, Indonesia; 34 Tilburg University, Tilburg, The Netherlands; 35 National and Kapodistrian University of Athens, Athens, Greece; 36 Faculty of Philosophy, University of Belgrade, Belgrade, Serbia; 37 Department of Psychology, University of Wah, Wah Cantt, Pakistan; 38 Vilnius University, Vilnius, Lithuania; 39 Zurich University of Applied Sciences, Zurich, Switzerland; 40 King Saud University, Riyadh, Saudi Arabia; 41 Universidad Nacional del Oeste, Ituzaingó, Buenos Aires, Argentina; 42 National Chengchi University, Taiwan, China; 43 University of Essex, Colchester, United Kingdom; 44 University of Brasilia, Brasília, Brazil; 45 University of Sussex, Brighton, UK; 46 Renmin University of China, Beijing, China; 47 The Education University of Hong Kong, Hong Kong, China; 48 Lingnan University, Hong Kong, China; 49 Faculty of Business, Hong Kong Polytechnic University, Hong Kong, China; 50 University of Georgia, Athens, US; 51 Graduate School of Education, Kyoto University, Kyoto, Japan; 52 King’s Business School, King’s College London, London, United Kingdom; 53 University of Iceland, Reykjavík, Iceland; 54 Baze University, Abuja, Nigeria; 55 Bahçeşehir University, İstanbul, Turkey; 56 University of Gdańsk, Gdańsk, Poland; 57 HUN-REN Institute of Cognitive Neuroscience and Psychology, Research Centre of Natural Sciences, Budapest, Hungary; 58 Department of Social and Intercultural Psychology, Károli Gáspár University of the Reformed Church, Budapest, Hungary

**Keywords:** Human behaviour, Society

## Abstract

This data paper presents a dataset, of 12,361 observations compiled from the Happiness Meanders project, which explores cultural variations in individual and family well-being, ideal and actual happiness, emotional experiences and expressions, and cultural models of selfhood across 48 countries. Participants were recruited through academic networks. Data were collected using standardised scales, including the Satisfaction with Life Scale, the Interdependent Happiness Scale, the Cultural Models of Selfhood Scale, and the Emotional Experience and Expression inspired by Affect Valuation Index. The dataset underwent thorough technical validation, including checks for variable consistency, handling missing data, and identifying potential response biases. A filter for data quality was applied, with potentially unreliable data flagged for exclusion. This dataset offers a valuable resource for examining cultural influences on emotional dynamics (frequence of experience and expression), individual and family oriented evaluations of happiness, ideal and actual evaluation of happiness, cultural models of selfhood, and can support further research in cross-cultural psychology and related social sciences.

## Background & Summary

The *Happiness Meanders* project was initiated in 2017. Between May 2017 and February 2019, data were gathered. The shared dataset^1^ includes 48 countries across six continents.

The Happiness Meanders project emerged from the observation that, since the 1980s, psychological research on well-being has largely privileged individually oriented conceptions of happiness^2,3^. This emphasis has contributed to the establishment of a dominant scientific narrative in which societal happiness appears consistently associated with what Triandis^4^ and Hofstede^5^ conceptualized as individualism^6,7^. This overarching narrative has been termed: *individualism-themed happiness*^8^. Departing from this comprehension, along with cross cultural research^9^, the Happiness Meanders project hypothesizes that when well-being is operationalized through more *collectivism-themed conceptions of happiness*, this association is attenuated. These findings suggest that there are multiple cultural ways of living a fulfilling and happy life, some emphasizing connection to others and group belonging, and others prioritizing the interests of the self.

These propositions are supported by two complementary distinctions. First, happiness evaluations differ in terms of who is taken as the referent of well-being, that is, the subject of measure, defining the focus of life satisfaction (individual vs. family). Second, they differ in terms of how happiness is evaluated, that is, the concept of happiness, which we label the orientation toward happiness (life satisfaction vs. interdependent happiness).

The focus of life satisfaction (subject of measure) refers to what people prioritize in their conception of happiness, namely whether happiness is primarily oriented toward the individual or toward the family. To capture this distinction, the operationalization relies on two independent sets of items based on the same phrasing. One scale is formulated from an individual-centered perspective, for instance: *“The conditions of your life are excellent”*^2^ (rated on a 9-point Likert scale). A second scale is formulated in a way that foregrounds the family as the focal referent: *“The conditions of your family’s life are excellent”* (rated on a 9-point Likert scale). This initial dialectical distinction allows a nuanced apprehension of the focus of life satisfaction, as it highlights the complexity of the referential frame through which happiness is evaluated (individual vs. family).

The orientation toward happiness (concept of happiness) captures a second dialectical variation by contrasting autonomy-based and heteronomy-based evaluative logics^10^. Life satisfaction items reflect an autonomy-oriented framework. They are grounded in an independence-oriented evaluative logic, in which happiness is assessed in relation to fulfilments along one’s life trajectory, hedonic experiences and personal achievements, as illustrated by the item: *“So far, you have gotten the important things you want in life”*^2^ (9 points Likert scale). By contrast, interdependent happiness items rely on a heteronomy-based framework, in which happiness is evaluated through interpersonal harmony, others’ well-being, a calm and serene emotional state, and the feeling of being just like others, as exemplified by the item: *“You believe that your life is just as happy as that of others around you”* (rated on a 9-point Likert scale^11^). Taken together, this dialectical distinction indicates that orientation toward happiness is structured by the interplay between autonomy and heteronomy, corresponding respectively to independent and interdependent conceptions of the self^7,12^.

**Fig. 1:**
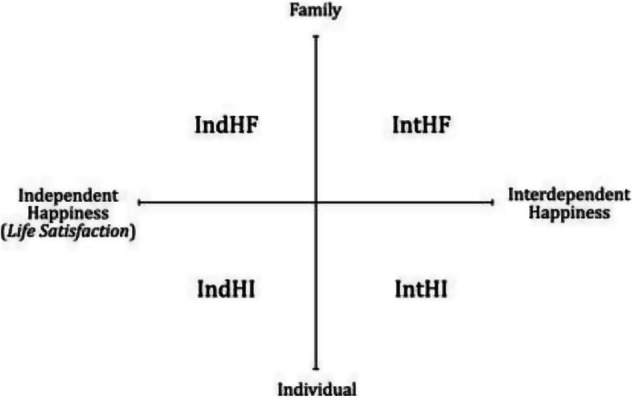
Four configurations of societal happiness within a two-dimensional framework. The vertical axis differentiates the subject of happiness (individual vs. family). The horizontal axis differentiates the concept of happiness (independent happiness-life satisfaction vs. interdependent happiness). The four quadrants represent ideal-typical configurations within a continuous positional space, in which countries can be located based on their mean scores on the corresponding scales.

Accordingly, the combination of the focus of life satisfaction (subject of measure: individual vs. family) and the orientation toward happiness (concept of happiness: independent happiness vs. Interdependent happiness) yields four broad configurations of societal happiness. These range from societies of independent happy individuals (IndHI) to societies of interdependent happy individuals (IntHI), independent happy families (IndHF), and interdependent happy families (IntHF). Figure 1 presents this conceptual framework.

As described above, the first part of the study focuses on what has been referred to as actual happiness, that is, participants’ evaluations of their own happiness. A second part is devoted to ideal happiness, which captures individuals’ conceptions of what happiness should ideally be. In this section, participants were asked to respond by adopting the perspective of an ideal or perfect person. Accordingly, the same scales^2,11^ were administered with the following instruction: *“This time, instead of answering how much these statements describe you, we would like you to indicate how much you think the ideal or perfect person would agree that each statement describes him or her.”* By including both actual and ideal evaluations of happiness within the same dataset^1^, the *Happiness Meanders* dataset^1^ enables the examination of different cultural features that shape happiness. (Table 1)

**Table 1:** Scales and Corresponding Lines in the Codebook File.

Scale	Corresponding line in the codebook file
SWLS-I(a)	From line 9 to 13
SWLS-F(a)	From line 131 to 135
IHS-I(a)	From line 14 to 22
IHS-F(a)	From line 136 to 144
Cultural Models of Selfhood Scale	From line 23 to 70
Emotional Experience and Expression Inventory	From line 71 to 130
SWLS-I(i)	From line 145 to 149
SWLS-F(i)	From line 159 to 163
IHS-I(i)	From line 150 to 158
IHS-F(i)	From line 164 to 172
Satisfaction with other life domains	From line 173 to 181
Sociodemographic items	From line 182 to 200

Extending these perspectives, the dataset^1^ also provides information on the frequency with which participants experience and express a wide range of positive and negative emotions. Participants reported on thirty distinct emotions, assessed separately for experience and expression, allowing for the examination of relationships between emotional experiences, happiness, and selfhood across cultural contexts. The initial selection of emotions was informed by research on ideal affect, operationalized through the Affect Valuation Index (AVI^13^). Drawing on the broader literature on cultural variation in emotions (e.g., Ekman^14^), the emotion list was subsequently expanded and adapted to capture a wider range of emotionally salient experiences across cultures (see Methodology). In particular, the original focus of the AVI was complemented by the inclusion of emotions considered psychologically fundamental and culturally salient^15^, such as being in love, which are not represented in the original framework.

Another key component of the dataset^1^ consists of measures assessing cultural models of selfhood. The questionnaire includes eight dimensions of cultural selfhood: seven dimensions proposed by Vignoles *et al*.^16^ as well as an additional dimension consisting in contextualised vs. decontextualized selfhood, incorporated specifically following recommendations by the scale’s author (see Methodology). The cultural models of selfhood^16^, inspired by research on independent and interdependent self-construals^17,18^, reflect how individuals perceive and define themselves in relation to others. The inclusion of this scale in the *Happiness Meanders* dataset^1^ allows for the examination of relationships between different types of happiness and emotional patterns.

The eight dimensions included in the dataset^1^ are: Consistency vs. Variability, Self-containment vs. Connectedness, Decontextualized vs. Contextualised, Difference vs. Similarity, Self-direction vs. Reception to influence, Self-expression vs. Harmony, Self-interest vs. Commitment to others, and Self-reliance vs. Dependence. Together, these dimensions allow for the examination of variation in cultural models of selfhood, ranging from more autonomous and independent orientations to more relational and interdependent orientations. The inclusion of these measures enables analyses of how different models of selfhood relate to patterns of happiness and emotional life across cultural contexts^19^.

The Happiness Meanders dataset^1^ was mainly designed to enable the examination of several questions central to cross-cultural research on happiness, selfhood, and emotional life. In particular, it allows researchers to investigate how happiness vary depending on the conceptualisation and measurement of happiness, including more interdependent-oriented approaches.

## Methods

### Questionnaire Design

Data were collected using a cross-sectional survey design. The *Happiness Meanders* questionnaire was developed following a multi-step procedure.

A complete list of all scales included in the questionnaire is provided below. The full English version of the questionnaire was archived and made publicly available via the Open Science Framework: https://osf.io/e8fgu (10.17605/OSF.IO/E8FGU).

The total number of items administered to participants was 182, grouped into 12 measurement instruments. The scales employed in the research included:

1. Satisfaction with Life Scale: Individual version (actual) – SWLS-I(a) – 5 items. This scale was used to assess actual individual satisfaction with life^2^ [e.g., “In most ways your life is close to your ideal”; rated on a 9-point Likert scale]
2. Satisfaction with Life Scale: Family version (actual) – SWLS-F(a) – 5 items. This scale was used and adapted to assess actual family satisfaction with life, inspired by Diener *et al*.^2^ [e.g., “In most ways the life of your family is close to ideal”; rated on a 9-point Likert scale]
3. Interdependent Happiness Scale: Individual version (actual) – IHS-I(a) – 9 items. This scale was used to assess actual individual interdependent happiness^20^ [e.g., “You believe that your life is just as happy as that of others around you”; rated on a 9-point Likert scale]
4. Interdependent Happiness Scale: Family version (actual) – IHS-F(a) – 9 items. This scale was used and adapted to assess actual family interdependent happiness inspired by Hitokoto & Uchida^20^ [e.g., “You believe that the life of your family is just as happy as that of other families around you”; rated on a 9-point Likert scale]
5. Cultural Models of Selfhood Scale – 48 items. Seven types of cultural models of selfhood were used^16^. One additional type was included: contextualized vs. decontextualized self^16,21,22^ [e.g., “If a close friend or family member is happy, you feel the happiness as if it were your own”; rated on a 9-point Likert scale]
6. Emotional Experience and Expression Inventory – 60 items. Thirty emotions were assessed through emotional experience (30 items) and emotional expression (30 items). This emotion set was inspired by the Affect Valuation Index (AVI^13^). However, fifteen culturally specific affective patterns were removed and replaced by fifteen emotions drawn from prior work on basic and culturally salient emotions^14,15^ [e.g., participants rate the frequency with which they experience and express emotions such as “calm”, “grateful”, or “in love”, each assessed separately on a 9-point scale ranging from “never” to “all the time”]
7. Satisfaction with Life Scale: Individual version (ideal) – SWLS-I(i) – 5 items. This scale was used to assess ideal individual satisfaction with life, inspired by Diener *et al*.^2^ [e.g., “So far you have gotten the important things you want in life”; participants indicated how much the ideal or perfect person would agree with each statement; rated on a 9-point Likert scale]
8. Satisfaction with Life Scale: Family version (ideal) – SWLS-F(i) – 5 items. This scale was used and adapted to assess ideal family satisfaction with life, inspired by Diener *et al*.^2^ [e.g., “In most ways the life of your family is close to ideal”; participants indicated how much the ideal or perfect person would agree with each statement regarding their family; rated on a 9-point Likert scale]
9. Interdependent Happiness Scale: Individual version (ideal) – IHS-I(i) – 9 items. This scale was used to assess ideal individual interdependent happiness inspired by Hitokoto & Uchida^20^. [e.g., “You make significant others happy”; participants indicated how much the ideal or perfect person would agree with each statement; rated on a 9-point Likert scale]
10. Interdependent Happiness Scale: Family version (ideal) – IHS-F(i) – 9 items. This scale was used and adapted to assess ideal family interdependent happiness inspired by Hitokoto & Uchida^20^. [e.g., “Your family makes significant others happy”; participants indicated how much the ideal or perfect person would agree with each statement regarding their family; rated on a 9-point Likert scale]
11. Satisfaction with other life domains – 9 items. This scale assesses satisfaction with nine areas of life (health, personal financial situation, family financial situation, achievements, relationship with family, relationship with friends, relationship with other people, capabilities, personal growth). [e.g., “You are satisfied with your relationship with family”; rated on a 9-point Likert scale]
12. Sociodemographic items – 9 items. [including age, gender, monthly budget, level of parental education, and living area (rural vs. urban)]

### Data Acquisition

The data collection period ranged from 2017 to 2019. Two data collection modalities were made available: paper-based and online surveys formats. Researchers were asked to select the modality most familiar to participants. Both a paper-based template and an online survey were provided to accommodate these preferences.

All measures used in the Happiness Meanders project were administered either in existing validated local language versions, or in newly translated versions. When validated local-language versions were not available, translations were produced using a back-translation procedure^23^. The English version of the scales was used as the foundation for all translations.

Participants were recruited using a convenience sampling strategy through academic networks and local collaborators, with a target sample size of approximately 200 participants per country. Actual sample sizes varied across countries due to local constraints and recruitment conditions. Collaborators were primarily recruited from previous large-scale cross-cultural projects^24,25^. Additional collaborators were identified through the International Association for Cross-Cultural Psychology and, in some cases, the European Association of Social Psychology. Some researchers joined the project following personal contacts, recommendations, or conference presentations.

Ethical approval for the study was obtained from the Ethics Board for Research Projects at the Institute of Psychology of the Polish Academy of Sciences (ERB approval number #7/11/2017). Additional approvals were obtained from local Institutional Ethic Committees where required. All participants provided informed consent, prior to participation. In addition, where applicable, the study protocol and the data-sharing agreement were duly approved by the ethics committees /IRBs.

As part of the participation agreement, informed consent was obtained from the participants. It was specified that the data would be shared in an aggregated form (i.e. « Will you please help by completing our survey? The study takes 25 minutes, is anonymous, and the results will be analysed and reported only in the aggregate form »).

The complete questionnaire, including all items and instructions is available in the PDF format in the project repository. A detailed codebook is provided, containing all variables, item wording, response scales and coding values.

## Data Records

The dataset^1^ is available at Open Science Framework (OSF) (10.17605/OSF.IO/E8FGU). The file names have been explicitly specified, and the variable names are provided in the nomenclature included in the HM codebook, HM_basic_syntax to upload, and Cleaning_data_syntax.”

The dataset^1^ includes 408 variables, each stored in a separate column, covering both directly measured and computed variables. A comprehensive description of these variables is provided in the Codebook file, which can be accessed in the repository. This dataset^1^ is accompanied by a script designed to calculate all selfhood-related variables.

The column ‘Label’ contains the specific question posed in the survey, i.e., the content of each survey item, while the ‘Values’ column delineates the range of values that the corresponding variable can take, according to the scale to which it belongs.

Regarding the codebook file, a clarification needs to be made. Specifically, the items corresponding to each scale in the codebook file are as follows:

As stated in the consent agreement and reflected in the dataset^1^, the data are fully anonymized. Consequently, they may be shared under the CC-BY license.

## Technical Validation

The process of technical validation was crucial to ensuring the consistency, accuracy, and integrity of the dataset^1^. All 50 country partial datasets^1^ were obtained in electronic form. The instruction for the researchers covered the proper instruction for digitising the data they obtained in paper form (see manual for collaborators uploaded to https://osf.io/e8fgu. Please note that only 48 of the dataset^1^ are available in the repository.

Each newly integrated dataset^1^ was systematically compared against the reference database to maintain standardisation. The validation process involved several key steps:

- Variable Consistency Check: The number of variables in each dataset^1^ was verified against the reference list to ensure complete coverage, correct sequencing, and consistent naming conventions. Any discrepancies were addressed to ensure the dataset^1^ is in line with the reference.
- Column Merging: In cases where a single variable had been fragmented across multiple columns, a merging procedure was applied to consolidate the information (e.g., for variable *education_parents* in case each parent was put into a separate column).
- Data Type Verification: All numerical variables were inspected to confirm that they were stored as numerical values. If any were encoded as strings, they were converted into the appropriate numerical format. In instances where text-based encoding was present in an unfamiliar language, an online translator was utilised to facilitate recoding into numerical values; in case of further doubts, the collaborating team was contacted to dispel these doubts.
- Handling of Missing Data: Missing values were systematically imputed using a placeholder value of 999 to ensure uniformity across datasets^1^.
- Value Range Validation: The validity of variable values was assessed to identify potential inconsistencies, such as entries outside predefined ranges (e.g., a variable restricted to values 1–7). Any erroneous values resulting from misclicks were corrected, while random or unclassifiable values were recoded as 999.
- Final Integration: Once cleaned and validated, each country’s dataset^1^ was appended to the aggregated database using structured merging procedures.

This validation framework ensured data coherence, minimised inconsistencies, and facilitated reliable cross-country comparisons.

A separate filter for data quality was later developed to ensure the accuracy and integrity of the data. The filter script can be found in the repository. A variable called data_quality was created to represent the outcome of running the script. This variable is already included in the dataset^1^, with a value of 0 indicating bad data quality and 1 indicating good data quality. Due to significant psychometric concerns, data from Bulgarian participants were excluded from the analysis and classified as low-quality. Following this exclusion, responses to the selfhoods and emotions sections of the questionnaire were systematically examined for patterns indicative of random or careless responding, including the phenomenon known as Christmas treeing. About Christmas treeing, it occurs when a respondent follows a predictable pattern, such as shifting answers incrementally (e.g., 5,6,7,8,7,6,5,6,7,8). To assess Christmas treeing, differences between adjacent response items were computed, quantifying the variation between consecutive answers. Next, the standard deviation of these differences was calculated. Participants for whom the standard deviation in differences equaled zero (i.e., when differences in their responses were always the same, e.g., either one or two or any other number of response points) were flagged as unreliable.

Additionally, to mitigate the potential risk of data falsification or fabrication, we utilised the ‘Identify Duplicate Cases’ function in SPSS. At the time of analysis, this function could process no more than 64 variables, so we applied it separately to three consecutive sections of the questionnaire: (1) individual well-being and cultural models of selfhood, (2) emotions, and (3) family and ideal well-being. If a participant was flagged as providing duplicate responses in any of these sections, their data were classified as low-quality.

Importantly, all data included in the main database remain accessible, allowing readers to independently assess data quality beyond the procedures we implemented and described above.

## Usage Notes: Further analytical opportunities

Although the Happiness Meanders dataset^1^ has already been used to test a range of hypotheses in cross-cultural psychology^8,9,19,26–30^, it has not yet been exhaustively explored. Given its breadth and structure, comprising 12 measurement instruments, 182 items, and 12,361 participants across 48 countries, the dataset^1^ supports many additional analytical approaches. The examples below are intended as illustrative rather than exhaustive research directions.

First, the gap between actual and ideal happiness has not yet been systematically examined, and could serve as a proxy indicator of cultural normative pressure across societies.

Second, the relationships between the eight dimensions of selfhood^16^ and the four configurations of societal happiness have not yet been explored, and examining their convergence could contribute to a more integrated understanding of cultural variation in happiness.

Third, the thirty emotions assessed separately for frequency of experience and expression have not yet been related to the four configurations of societal happiness, and doing so could connect this dataset to broader research on ideal affect^13^.

Beyond these directions, the dataset lends itself to more focused uses. Researchers interested in a specific cultural comparison could selectively draw on data from two or more countries, without engaging the full breadth of the dataset.

## Data Availability

Like mentioned in Data Record, the dataset^1^ is available at Open Science Framework (OSF) (10.17605/OSF.IO/E8FGU). The file names have been explicitly specified, and the variable names are provided in the nomenclature included in the HM codebook, HM_basic_syntax to upload, and Cleaning_data_syntax.”

## Code availability

We provide an SPSS script for the calculation of selected variables. This code is available in the repository. 
